# SPAMRI: A MATLAB Toolbox for Surface-Based Processing and Analysis of Magnetic Resonance Imaging

**DOI:** 10.3389/fnhum.2022.946156

**Published:** 2022-07-07

**Authors:** Zhiliang Long

**Affiliations:** Sleep and NeuroImaging Center, Faculty of Psychology, Southwest University, Chongqing, China

**Keywords:** morphology, surface measurements, user-friendly GUI, SPAMRI, quality check

## Abstract

Structural magnetic resonance imaging (MRI) has elicited increasing attention in morphological surface studies due to its stability and sensitivity to neurodegenerative processes, particularly in exploring brain aging and psychiatric disease. However, a user-friendly toolbox for the surface-based analysis of structural MRI is still lacking. On the basis of certain software functions in FreeSurfer, CAT and ANTs, a MATLAB toolbox called “surface-based processing and analysis of MRI” (SPAMRI) has been developed, which can be performed in Windows, Linux and Mac-OS. SPAMRI contains several features as follows: (1) open-source MATLAB-based package with a graphical user interface (GUI); (2) a set of images that can be generated for quality checking, such as Talairach transform, skull strip, and surface reconstruction; (3) user-friendly GUI with capabilities on statistical analysis, multiple comparison corrections, reporting of results, and surface measurement extraction; and (4) provision of a conversion tool between surface files (e.g., mesh files) and volume files (e.g., NIFTI files). SPAMRI is applied to a publicly released structural MRI dataset of 44 healthy young adults and 39 old adults. Findings showed that old people have decreased cortical thickness, especially in prefrontal cortex, relative to those of young adults, thereby suggesting a cognitive decline in the former. SPAMRI is anticipated to substantially simplify surface-based image processing and MRI dataset analyses and subsequently open new opportunities to investigate structural morphologies.

## Introduction

Structural magnetic resonance imaging (MRI) is a non-invasive technique and has been widely used because of its high-contrast sensitivity and spatial resolution and allowance for the accurate measurement of brain structures. MRI is considered a powerful tool for investigating the anatomy and pathology of the brain (He et al., 2007; Hibar et al., 2016; Tessitore et al., 2016; Magon et al., 2018; Matsuo et al., 2019). Currently, volume and surface measurements are widely used in brain morphometric studies.

Volume-based morphometric measurement is an important aspect of voxel-based morphology (VBM) (Ashburner and Friston, 2000, 2001; Mechelli et al., 2005). VBM analysis calculates the gray/white matter concentration or absolute volumes of a voxel according to unmodulated or modulated methods, respectively. It has wide application in exploring aging and brain diseases (Good et al., 2001; Matsuda, 2013; Hibar et al., 2016; Gennatas et al., 2017; Matsuo et al., 2019). However, the interpretation of VBM results is somewhat ambiguous. At the basic level, group differences in gray matter volume are not only caused by independent changes in surface area and cortical thickness but also by the degree and pattern changes of folding. Therefore, changes in gray matter volume do not specifically correspond to any single morphological change.

Surface-based morphometric measurements, including those for cortical thickness, surface area, and folding, are computed on the basis of the cerebral cortex (Fischl and Dale, 2000). Each of these measurements is governed by different developmental processes (Wierenga et al., 2014). For example, surface area expansion can be associated with increased number of cortical columns (i.e., referring to a group of neurons that encode similar features, and these columns are oriented perpendicularly to the cortical surface), whereas increased cortical thickness can be assumed to be related to changes in the neuronal microcircuitry within a column (Fjell et al., 2015). Furthermore, thickness and surface area are heritable than folding (Panizzon et al., 2009). Thus, changes in cortical thickness can be regarded a useful surrogate of patterns of microstructural differences. Studies on aging and brain diseases have used surface measurements to characterize the trajectory of brain microstructural changes and pathology of brain disturbances, respectively (Good et al., 2001; Shaw et al., 2008; Matsuda, 2013; Hong et al., 2014).

Several algorithms were developed to determine these surface measurements e.g., the cortical thickness. A surfaced-based method that modeled the cerebral cortex as a surface mesh was proposed to compute cortical thickness, which was available in the popular FreeSurfer software (Dale et al., 1999; Fischl et al., 1999; Fischl, 2012) (https://surfer.nmr.mgh.harvard.edu/fswiki). Alternative methods such as the Laplace's Equations (Jones et al., 2000) or registration-based solution (Das et al., 2009) have also been developed for cortical thickness computation, which were employed in ANTs software (Avants et al., 2014). However, processing and analyses employed by these packages are conducted by subject-by-subject command lines. Furthermore, several manual procedures were needed, which may be time consuming. This can increase the probability of inadvertent mistakes. Thus, a user-friendly graphical user interface (GUI) for surface-based imaging processing and analysis is necessary, especially for clinicians and MRI beginners.

In the current study, we have developed a MATLAB toolbox called “surface-based processing and analysis of MRI” (SPAMRI) based on certain software functions of FreeSurfer, CAT, and ANTs. This toolbox provides processing steps in parallel, and generates a number of images for quality checking. It also provides GUI for statistical analysis, multiple comparison correction, reporting of results, and extraction of surface measurements. In addition, SPAMRI can be used to perform data-type conversion between surface mesh files and volume-based files.

## Materials and Methods

SPAMRI, which has been developed by using MATLAB (R2014b) with Windows (which needs windows subsystem installed), Linux and Mac-OS, calls several processing functions in FreeSurfer, CAT, and ANTs. In this paper, the processing in SPAMRI is initially described, and then an introduction to realize each aforementioned procedure is presented.

**Figure 1:**
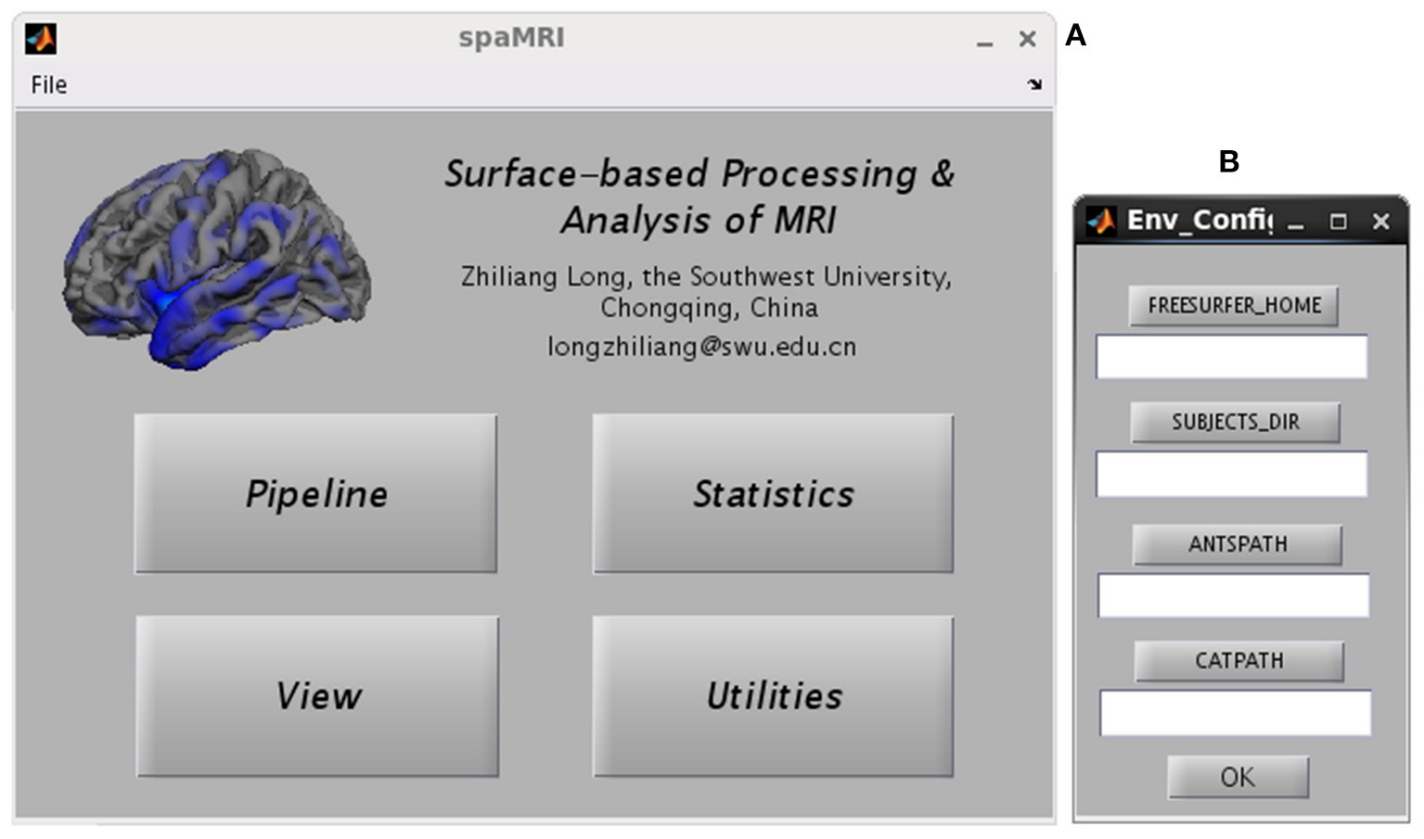
Graphical user interfaces of SPAMRI **(A)** and environment configuration **(B)**. The main window of SPAMRI includes four panels: Pipeline, Statistics, View and Utilities.

**Figure 2:**
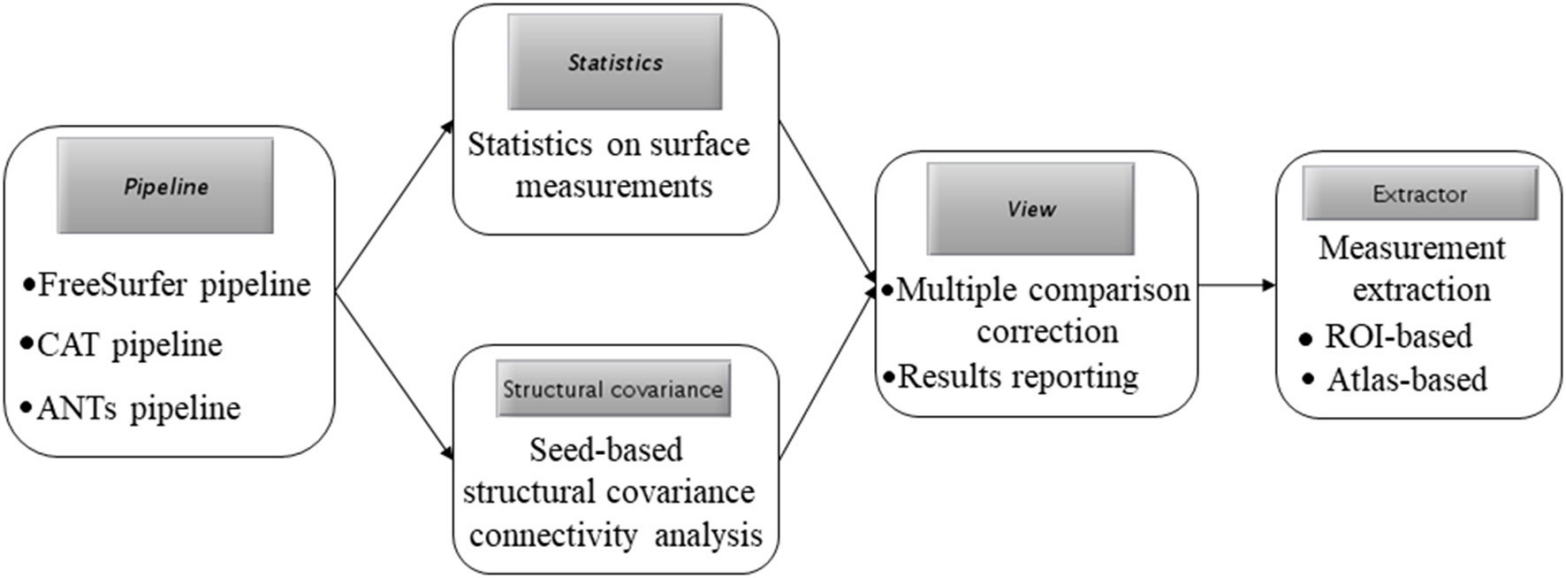
The flow chart of SPAMRI.

Figure 1A presents the main GUI of SPAMRI that includes four panels: (1) pipelines involved in cortical reconstruction and surface measurement calculation; (2) statistical analysis; (3) multiple comparison correction and result reporting; and (4) extraction of surface measurements. Four parameters, namely, FREESURFER_HOME, ANTSPATH, CATPATH and SUBJECTS_DIR, are needed before running SPAMRI. The first three parameters indicate the location where the FreeSurfer, ANTs and CAT are installed, and the last denotes the location where output files are saved (Figure 1B). As indicated by the flow chart of SPAMRI (Figure 2), these panels are introduced in detail in subsequent sections.

### Pipeline

**Figure 3:**
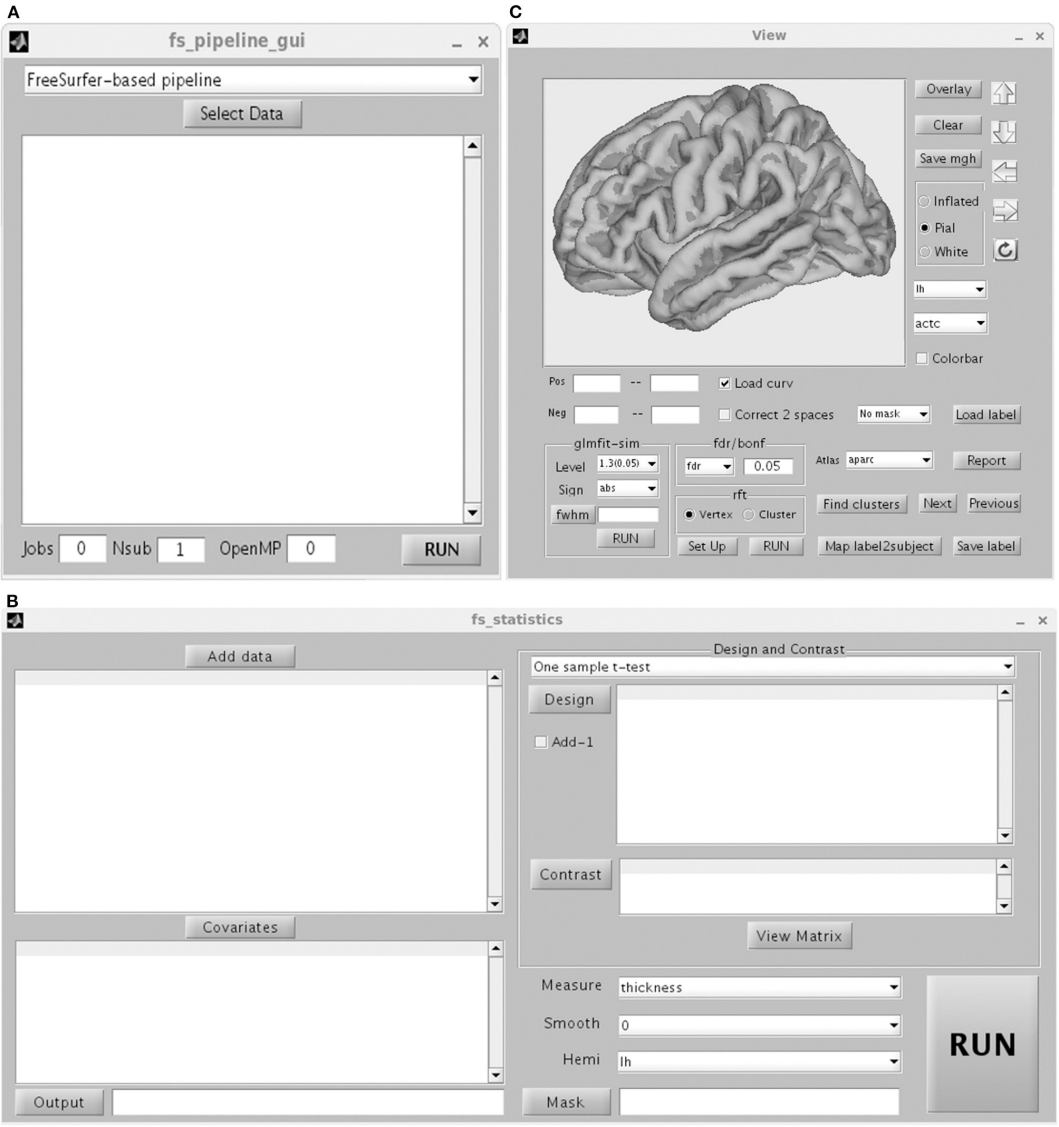
Graphical user interface of Pipeline **(A)** including FreeSurfer-based pipeline, ANTs-based pipeline and CAT-based pipeline, statistics **(B)** and view **(C)**.

Figure 3A shows the GUI of pipeline. The *Freesurfer-based pipeline* in current toolbox was a little different from the processing steps in FreeSurfer software (https://surfer.nmr.mgh.harvard.edu/). The constructed white matter surface and gray matte surface in FreeSurfer were highly depended on the brain image without skull, especially for the structural image with low resolution. Thus, in SPAMRI, a segmentation procedure was employed firstly to obtain gray matter, white matter and cerebrospinal fluid, which were then combined to generate a brain mask. The resulted brain mask was used to mask the structural image, resulting in a cleanly brain image without skull. This brain image with no skull was used as the input of “recon-all” pipeline in FreeSurfer. The following steps mainly included volumetric labeling, white matter segmentation, white matter/pial cortical reconstruction, spherical registration, cortical parcellation, cortical thickness estimation, resampling cortical measurement maps into standard (fsaverage) space and smooth.

The *ANTs-based pipeline* (http://stnava.github.io/ANTs/) included template construction, image registration, bias correction, tissue segmentation and cortical thickness estimation based on the diffeomorphic registration. Additionally, a CAT-based cortical thickness computation pipeline (Gaser and Dahnke, 2016) was included, in order to perform comparative studies.

There are two kinds of data import for all the three pipelines: (1) just import the T1 images of all subjects; (2) import the folder (e.g., named T1Raw) that contains subject folders (e.g., sub001, sub002, …). The T1 image of each subject is in the corresponding subject folder.

The GUI can also perform parallel computation, and thus, several subjects can be processed simultaneously. The description on how to install the parallel computation function can be found in this toolbox. The procedures can be conducted in parallel by using the “-openmp” function within a subject process.

### Quality Control

**Figure 4:**
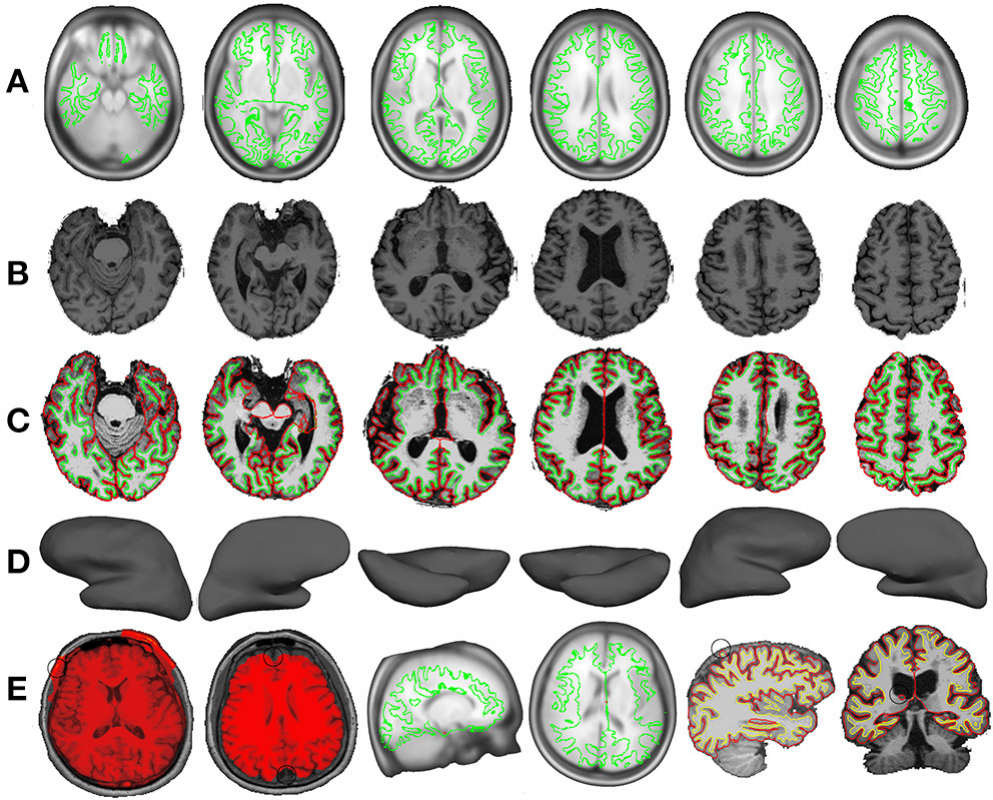
A set of pictures for checking the quality of talairach transform **(A)**, skull strip **(B)**, surface reconstruction **(C,D)**. The illustration of “bad” quality **(E)** includes skull strip failure (donated by black circles), talairach transform failure and surface construction failure (indicated by black circles).

Quality control is a necessary step to make sure that the results are not biased and interpretable. The *Freesurfer-based pipeline* in SPAMRI will generate one picture for each subject, in order to check the quality of data processing. The quality control includes (1) skull stripping (Figure 4B). Removal of skull is critical for surface construction. The skull stripping in SPAMRI is based on image segmentation. Thus, segmentation failure would result in removing the skull incorrectly. If in this case, choosing another segmentation algorithm or using “bet” in FSL would help; (2) talairach registration (Figure 4A). This is the talairach transform that maps the image from subject native space to standard space. Manual edition is needed if the talairach transform fails; (3) surface construction (Figures 4C,D). Manual fixation is also needed if the white matter surface and gray matter surface are constructed incorrectly. For example, voxels of white matter or cerebrospinal fluid are identified as gray matter voxels. The details of manual fixation can be seen in the FreeSurfer website (https://surfer.nmr.mgh.harvard.edu/). The illustration of “bad” data processing quality can be seen in Figure 4E.

### Statistics

The procedure for statistical analysis involves the calling of general linear model (Figure 3B). In SPAMRI, users only need to import data (mgh files resulted from FreeSurfer-based pipeline or gii files from CAT-based pipeline) and select several parameters to generate the statistics. It doesn't work with ANTs-based pipeline currently, because the cortical thickness map resulted from ANTs-based pipeline was voxel-based instead of surface mesh-based. Statistics on these maps can be performed using other toolboxes such as SPM (https://www.fil.ion.ucl.ac.uk/spm/), FSL (https://fsl.fmrib.ox.ac.uk/fsl/fslwiki) and AFNI (https://afni.nimh.nih.gov/).

#### One-Sample t-Test

This test verifies the hypothesis by which the mean cortical measurement across the group is equal to a specific value. Measurements for thickness, area, area_pial, sulc, curv, pial_lgi, volume, jacobin_white, w-g.pct, white.H, and white.K (Fischl and Dale, 2000) are included in this test. The design matrix and contrast matrix were determined manually, just simply “telling” the toolbox which subjects belong to one group, which subjects belong to another group. The “Run” button is pressed to perform statistical analysis after viewing the design matrix of the general linear model fitting. A mask can be added to restrict the procedure within a limited brain cortical area. By default, statistical analysis is conducted for either the entire left cerebral cortex or the entire right cerebral cortex. A covariate can be added to regress out the effect of a variable of no-interest.

#### Two-Sample t-Test

This test verifies the difference in cortical measurements between two groups, such as patient vs. control.

#### Multiple Regression

This test verifies the linear relationship of cortical measurement with behavioral or clinical score in one group.

#### One-Way ANOVA

Currently, one-way ANOVA only tests the between-subject effects of cortical measurements across three or more groups.

#### Two-Way ANOVA

The results of between-subject two-way ANOVA, depict the main effect of each factor and the interaction effect between two factors.

### Multiple Comparison Correction and Reporting of Results

The “View” GUI presents the statistical map, performs multiple comparison correction, and reports the statistical results (Figure 3C). Any type of mgh or gii file is allowable. However, statistical analysis should be conducted for the left or right hemisphere.

SPAMRI provides four methods of multiple comparison correction: (1) Bonferroni. Assume that the corrected *p* is 0.05. If the statistical levels of vertices are <0.05/*N*, where the *N* is equal to 163,842 for the left and right hemispheres in standard space, then the vertices survive Bonferroni correction. (2) False positive rate (FDR). The FDR procedure was programmed at the vertex level based on the MATLAB function “lme_mass_FDR” (Genovese et al., 2002), which has been implemented in FreeSurfer. It controls the fraction of detected vertices that are false positives. (3) Monte Carlo simulation (Hagler et al., 2006). This method initially synthesizes a z map. This z map is subsequently smoothened and operated in threshold (with a primary p threshold) at the vertex level, as a consequence of spatial clusters generated. Then, the maximum cluster size is recorded. The procedure is repeatedly iterated by the thousands (usually >5,000), resulting in a distribution with maximum cluster size. The same threshold is applied to the original data, and data clusters are obtained. Then, the significant level of each cluster is determined according to the proportion of values that exceed the cluster size in the distribution. To apply this method, three parameters are needed: cluster-forming threshold (e.g., a threshold of three corresponds to a *p* value of 0.001), the sign (positive, negative, or absolute), and the estimated FWHM. The procedure was coded based on the function of “mri_surfcluster” in FreeSurfer.

These correction methods can be also conducted in Qdec or Freeview (https://surfer.nmr.mgh.harvard.edu/fswiki/FreeviewGuide) in the FreeSurfer software. However, unlike Qdec/Freeview, the current toolbox provides another correction method that is based on random field theory (RFT). RFT (Hayasaka et al., 2004; Hagler et al., 2006) is programmed on the basis of functions obtained from the SurfStat toolbox (Worsley et al., 2009) (http://www.stat.uchicago.edu/faculty/InMemoriam/worsley/research/surfstat/). In functional MRI studies, Gaussian RFT is common for multiple comparison correction. However, a priori assumption that the noise component of the image data is isotropic is needed. This assumption is not reasonable for cortical surface data due to the different amounts of surface stretching. Thus, the data should be warped into a space where the data is regarded isotropic before applying RFT in the surface data (Worsley et al., 1999). The RFT method can be conducted at vertex or cluster levels, which suggest the significance of the vertices or clusters. Vertex- and cluster-wise RFTs have been coded in SPAMRI to handle *T*-statistics and *F*-statistics. Furthermore, SPAMRI can adjust the *p* values for the left and right hemispheres.

After conducting multiple comparison correction, the statistical results can be generated by simply selecting an appropriate cortical atlas (the default is ?h.aparc.annot) and pressing the “Report” button. The information of all clusters is included in the report: such as statistical value of peak vertex, index of peak vertex, cluster size, number of vertex, Montreal Neurological Institute (MNI) coordinates of peak vertex, location of peak vertex, and cluster locations.

The “View” GUI can also save clusters as masks for the measurement extraction of the region of interest (ROI) by performing the following steps: press the “Find clusters” button; select a cluster for the ROI; and press the “Save label” button to save the cluster information. The GUI can also map the ROI from fsaverage space back to subject native space by using the “Map label2subject” function.

### Measurement Extraction and Calculator

**Figure 5:**
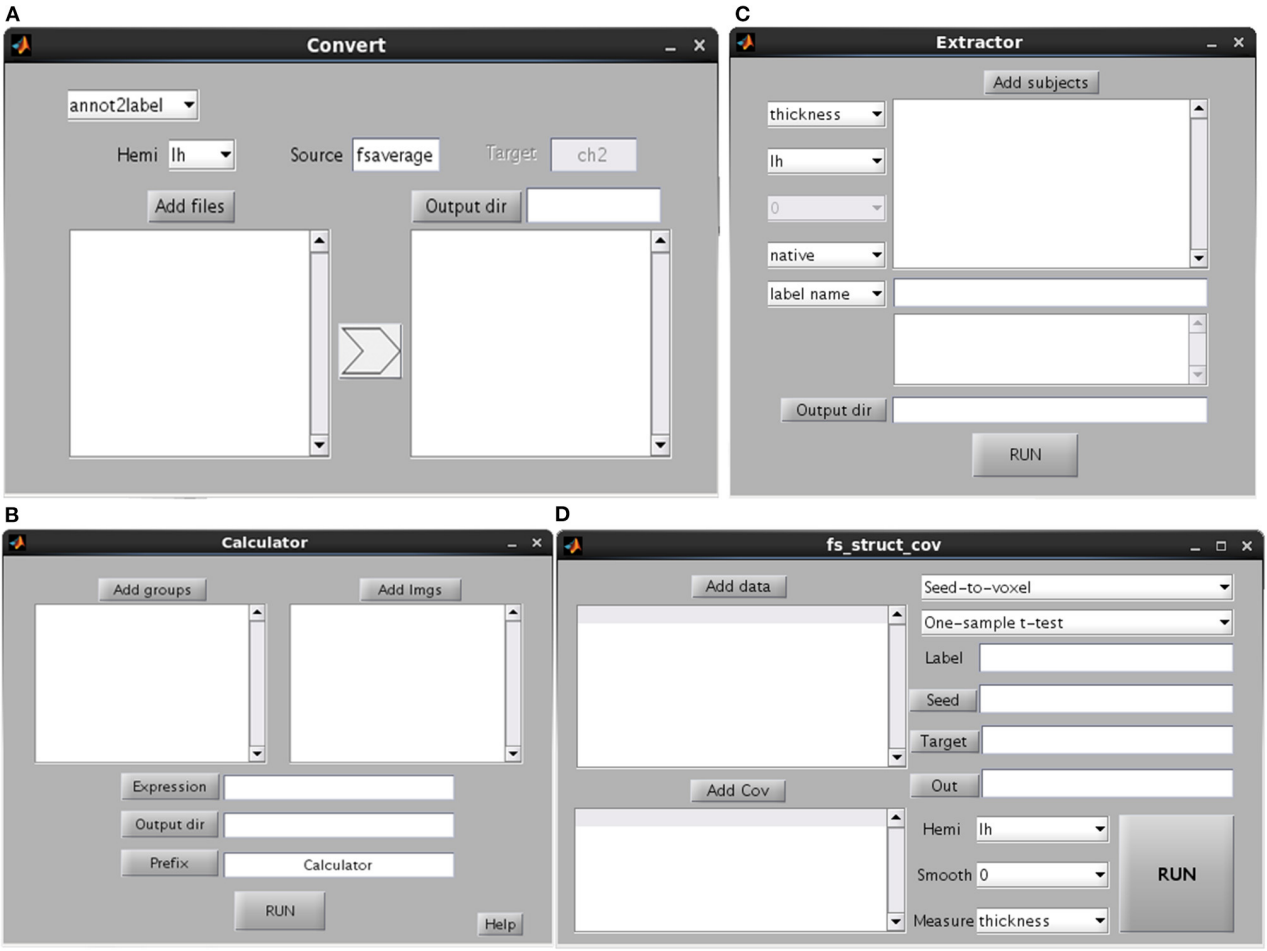
Graphical user interfaces of convert **(A)**, calculator **(B)**, extractor **(C)** and structural covariance connectivity analysis **(D)**.

This GUI is used to extract the cortical measurements of specific ROIs or cortical atlas (Figure 5C). Users can select whether the procedure will be conducted in subject native space or fsaverage space. If the procedure is conducted in native space, then the ROI/atlas file names need to be separated by comma. Otherwise, the ROI/atlas files in fsaverage space are selected for measurement extraction. The procedure of *Calculator* is used to perform mathematical operations on ROI files, e.g., the combination of a number of ROIs (Figure 5B).

### Convert

This GUI involves the conversion of different data types (e.g., between surface and volume data) (Figure 5A). The source is the subject space where the input files are obtained (the default is fsaverage), whereas the target is the subject space where the input files are mapped. Several kinds of conversions are listed. The “annot2label” divides a cortical atlas into different ROIs. The “label2annot” is used to merge a number of ROIs into a cortical atlas. The “label2label”/“surf2surf” is used to map ROI/surface files from one subject space (source) to another (target). The “label2nifti” is used to convert a surface ROI file into NIFTI file (i.e., generally used in SPM or FSL). It's useful in the case that a surface cluster needs to be further analyzed for volume-based studies. The default target subject is ch2, which is obtainable by performing recon-all steps on ch2.nii file. The “nifti2label” is utilized to map a volumetric ROI to surface ROI. The “surf2nifti” is employed to map surface files into NIFTI files. The “nifti2surf” is applied to map NIFTI files into surface files. The “mgz2nifti” is used to convert volumetric data (postfix with mgz) into NIFTI data. The “fs2gii” and “gii2fs” are to convert surface data between different types such as gii and mgh files.

### Structural Covariance Connectivity Analysis

Brain connectivity analysis has been widely applied in neuroscience and been programmed in many tools such as the rsHRF (Wu et al., 2021) and the conn (Whitfield-Gabrieli and Nieto-Castanon, 2012). The tool of SPAMRI (Figure 5D) also performs a seed-based covariance connectivity analysis with volumetric data (e.g., NIFTI file) or surface data (gii or mgh file) as input. The detailed procedures are as follows. For each group, the seed-to-whole brain covariance connectivity map is firstly constructed across participants. The statistics on the covariance connectivity maps are then performed by employing the SurfStat toolbox. Other kinds of structural covariance analysis such as ROI-to-ROI covariance connectivity analysis and causal analysis of structural covariance network can be seen in other tools, e.g., the BCCT (Xu et al., 2021).

### Testing the Effect of Age on Cortical Thickness by Using SPAMRI

Data were obtained from the Stockholm Sleepy Brain study (https://openneuro.org/datasets/ds000201/versions/00004), which included 86 young and old adults. The subjects were scanned twice before and after partial sleep deprivation. We selected the data of 83 participants (39 old adults [65–75 years] and 44 young adults [20–30 years]) before sleep deprivation (the T1 data of three participants were missing). All are right-handed and had no history of neurological psychiatric disorders. The young and old groups did not differ statistically in terms of gender. Written informed consent was obtained from each participant. The study was approved by the Regional Ethics Review Board of Stockholm (2012/1870-32). The detailed information have been reported in other papers (Nilsonne et al., 2017; Tamm et al., 2017).

MRI data were acquired with General Electric Discovery 3 T MRI scanner. T1-weighted structural images with whole-head coverage were obtained by using sagittal BRAVO sequence, TR = 6.4 s, TE = 2.8 s, voxel size=1 × 0.47 × 0.47 mm^3^, and flip angle = 11°.

#### Data Processing

The data were processed by using the current toolbox. Briefly, non-brain tissues were extracted from individual T1 images *via* hybrid watershed/surface deformation following intensity normalization (Segonne et al., 2004). The gray and white matters were segmented and subsequently used for cortical reconstruction. Then, the white (defined as gray/white boundary) and pial surfaces (defined as gray/CSF boundary) were identified. Cortical thickness was calculated as the closest distance from the gray/white boundary to the gray/CSF boundary at each vertex. Several images were generated to evaluate the quality of Talairach transform, brain image without skull, and surface reconstruction. The individual cortical thickness maps cannot be compared because they have different numbers of vertex. Thus, those maps were warped and registered as average spherical space (fsaverage) to optimally align sulcus and gyrus patterns, which then enabled the matching of cortical locations across entire surfaces and among individuals. The registered cortical thickness maps in fsaverage space were obtained for each hemisphere and smoothened by using 10-mm FWHM for statistical analysis.

#### ROI Analysis

We first validated the “Extractor” GUI by performing ROI analysis to investigate whether the mean cortical thickness across the entire cortical surface differed between young adults and old adults. Then, the mean cortical thickness values of the groups were extracted and compared by conducting two-tailed two-sample *t*-test.

#### Vertex-Wise Statistical Analysis

To test the age effect on cortical thickness at the vertex level, two-tailed two-sample *t-*test was conducted to determine the cortical thickness of the young adult group and the old adult group by using the “Statistics” GUI in the current toolbox. Multiple comparison correction was performed by using the “View” GUI. The no-threshold statistical map and corrected statistical maps were presented on the basis of the four aforementioned correction methods. The corrected *p* value of the vertex-level correction (including Bonferroni, FDR, and vertex-wise RFT) was set to *p* < 0.05. The cluster-forming threshold of the cluster-wise correction (including Monte Carlo simulation and cluster-wise RFT) was set to *p* < 0.001. The corrected *p* value of the clusters was set to *p* < 0.05. All methods were subjected to two-tailed test. The RFT procedure was also performed by using SurfStat toolbox (seen in Supplementary material).

## Results

The computer used for data-processing has 16G memory with eight-core CPU. The running time of T1 image processing of one subject was about 0.34, 12 h and over 24 ho in CAT, FREESURFER, and ANTs, respectively.

**Figure 6:**
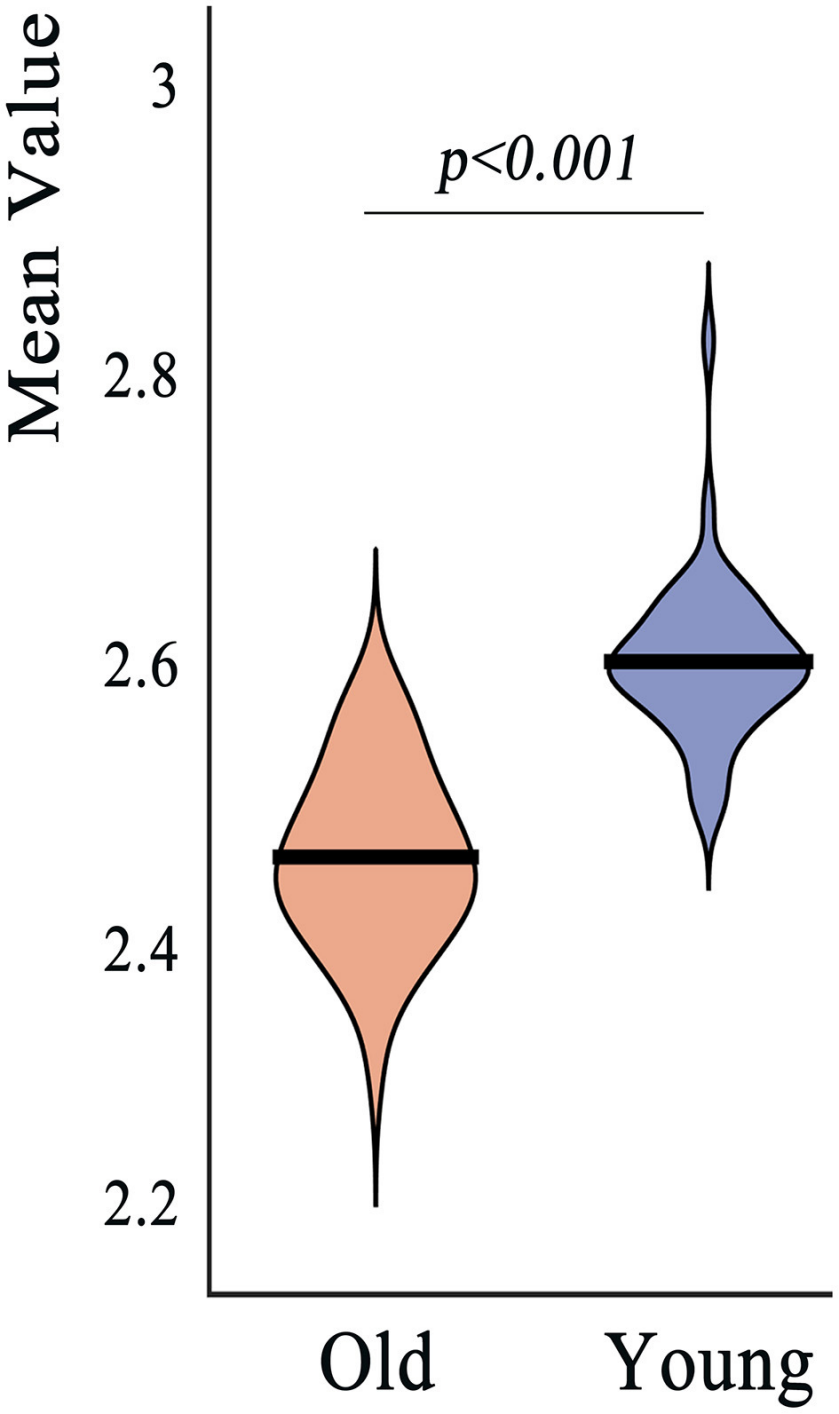
Region of interest analysis revealed significant decrease in mean cortical thickness across whole cerebral cortex in old adults compared to young adults.

**Figure 7:**
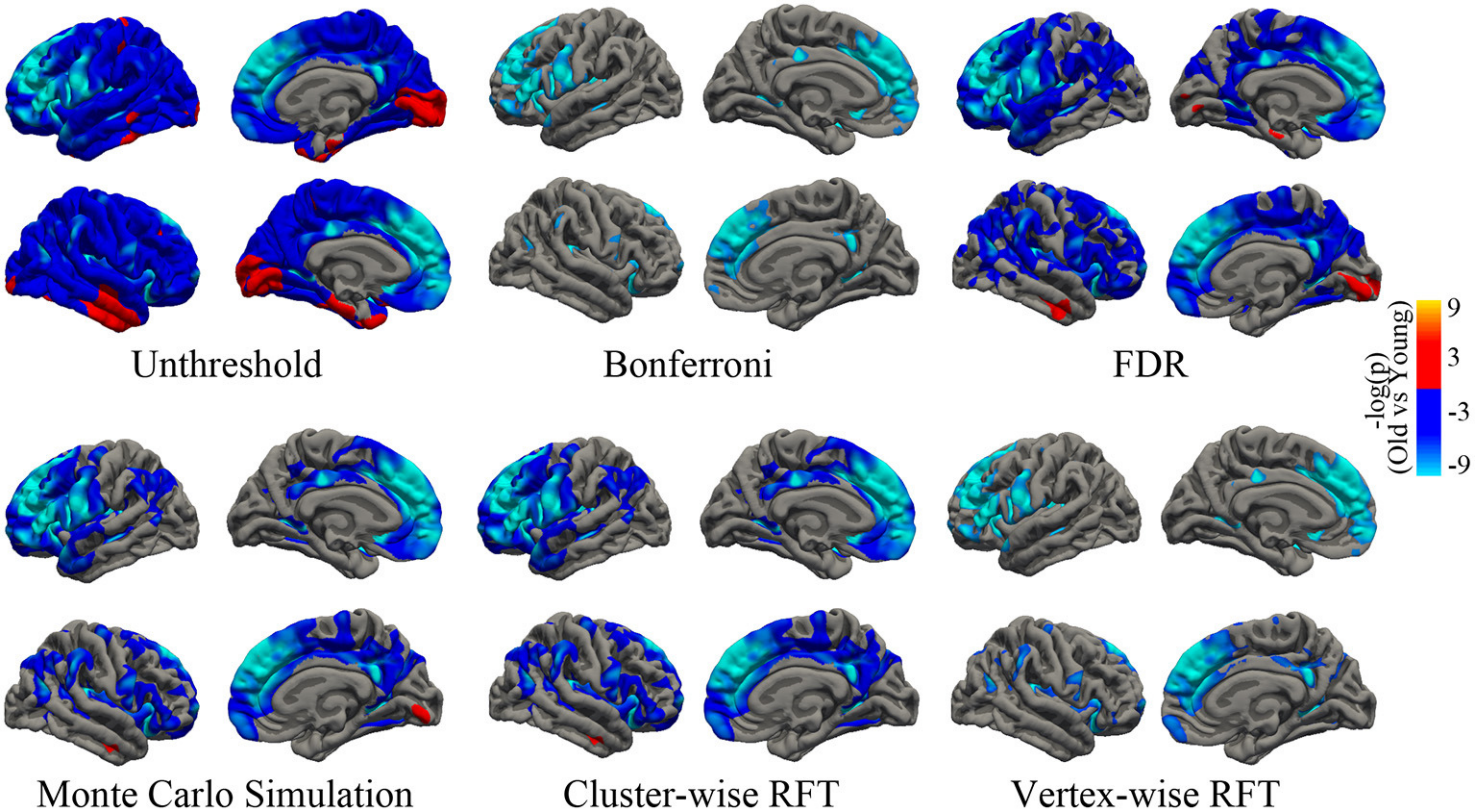
Unthresholed statistical map and thresholded maps using bonferroni, FDR, Monto Carlo simulation, cluster-wise/vertex-wise random field theory. The corrected statistical level was *p* < 0.05. The cluster-forming threshold was *p* < 0.001. Regions with blue/red color indicate deceased/increased thickness in old adults, respectively. FDR, false positive rate.

**Figure 8:**
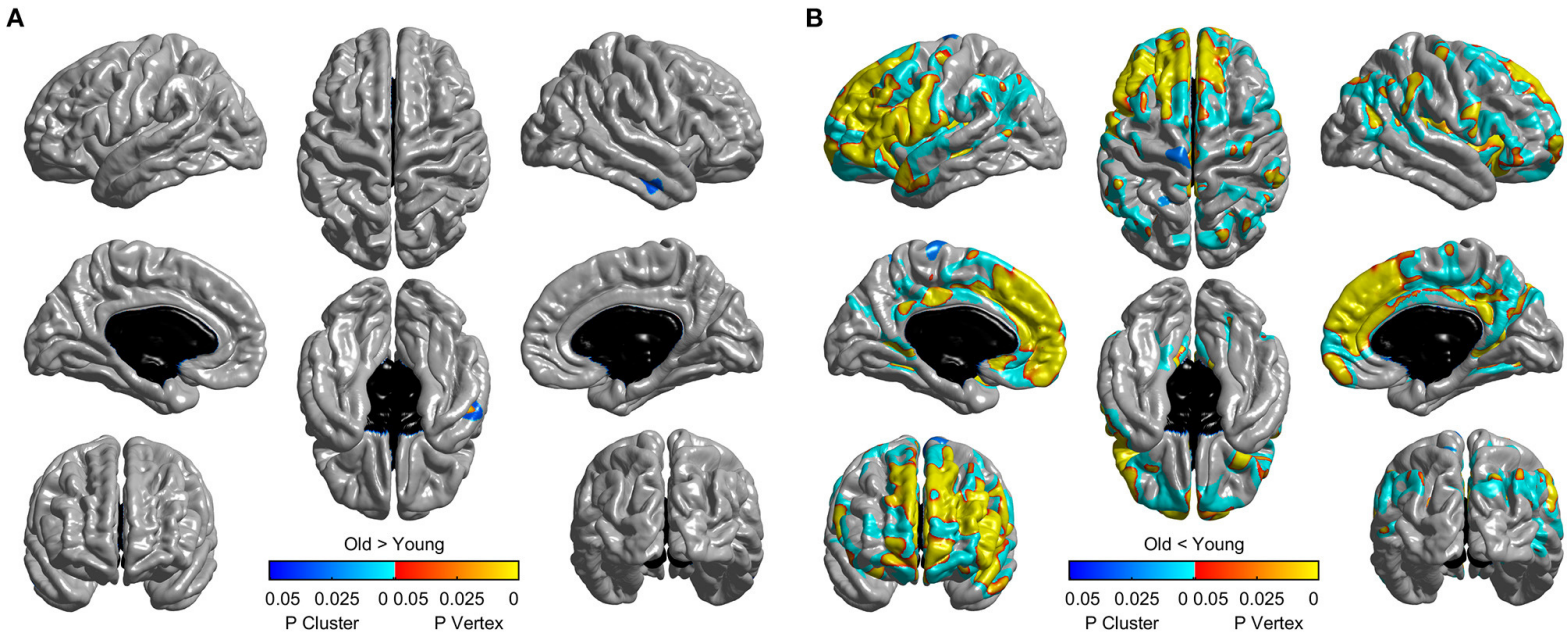
Thresholded statistical map using random field theory implemented in Surfstat. It tests the effect of “Old > Young” **(A)** and “Old < Young” **(B)** separately. Brain areas with warm/cool color survived vertex/cluster-level random field theory correction. The default cluster-forming threshold was *p* < 0.001.

The mean cortical thickness across whole cortex in old adults was significantly decreased compared with young adults (mean of old adults: 2.44 mm, mean of young adults: 2.59, *p* < 0.001, Figure 6). The difference was also significant when the total intracranial volume was included as a covariate. Vertex-wise statistics showed that old adults had decreased cortical thickness mainly in prefrontal cortex, anterior cingulate cortex, posterior cingulate cortex and several temporal regions, compared with young adults (Figure 7). It was worth noting that statistics using SurfStat detected more brain areas showing decreased cortical thickness in old adults (Figure 8).

## Discussion

The current work provides a MATLAB toolbox for surface-based processing of MRI. The toolbox can integrate data preprocessing, statistical analysis, and multiple comparison correction functions, and it offers convenient utilities, such as surface measurement extraction.

SPAMRI, which provides parallel functions for data preprocessing and simultaneous analysis of many subjects, has the potential to reduce the time-consumption. Removing brain skull is a crucial step in cortical construction and thickness estimation. In current toolbox, the skull was removed by performing segmentation on the structural image, which might improve the cortical construction. In addition, the quality of Talairach transform, skull strip, and cortical construction can be easily checked, and time can be saved by generating a set of images by using the current toolbox.

SPAMRI also provides the GUIs for statistical analysis and multiple comparison correction. The correction methods of Bonferroni, FDR, and Monte Carlo simulation can be validated by using FreeSurfer tools, such as Qdec or command lines. In addition, this toolbox provides a correction method for RFT. Although the RFT procedure in SPAMRI has been programmed on the basis of the SurfStat toolbox, they eventually differ in terms of testing and analysis. The RFT correction for T-statistic in SurfStat is performed by using one-tailed statistics (which may increase the false positive rate), whereas that in SPAMRI is conducted by using two-tailed statistics. As we can see from Figure 8, unlike the two-tailed vertex-wise RFT procedure in SPAMRI, the procedure in SurfStat detected more brain areas. For example, the middle temporal cortex shows increased cortical thickness in the old adult group (Figure 8A).

Specifically, the current toolbox revealed that older adults have significantly thinner mean cortical thickness than young adults, which accords with previous findings (Fjell et al., 2009; Hogstrom et al., 2013). The cortical thickness in the prefrontal cortex (including superior frontal cortex, rostral middle frontal cortex, laterior/medial orbitofrontal cortex, and rostral anterior cingulate cortex) of old adults is significantly thin. The patterns of regional cortical thinning due to aging have also been demonstrated by previous reports (Fjell et al., 2009; Thambisetty et al., 2010). Cortical thinning in the frontal cortex may indicate a decline in the cognitive ability of old people, and this observation is consistent with those in many morphological studies (Staff et al., 2006; Karama et al., 2014; Gennatas et al., 2017). Old people perform poorly in terms of executing processing tasks, such as memory tasks, which is presumed to be associated with the prefrontal cortex (Salat et al., 2002).

Currently, the structural covariance analysis between distributed brain regions becomes more and more popular (Alexander-Bloch et al., 2013). The structural covariance characterizes the co-variation of morphological changes between brain areas. For example, individuals with higher cortical thickness of Broca's area have also higher cortical thickness of Wernicke's area (Lerch et al., 2006). Usually, the structural covariance connectivity was constructed across participants at group level (Alexander-Bloch et al., 2013). However, it can't correlate the structural covariance with behavior or clinical scores. Thus, some authors developed individual structural covariance analysis (Tijms et al., 2012; Liu et al., 2021). More recently, the structural similarity analysis was proposed at individual level by combining multi-modal neuroimages (Seidlitz et al., 2018) and has been applied in many psychiatric disorders (Morgan et al., 2019; Li et al., 2021). The surface measurements computed from the current toolbox can be further used to conduct the structural covariance analysis by employing either the SPAMRI or other toolboxes, e.g., the BCCT (Xu et al., 2021).

In conclusion, SPAMRI is a user-friendly toolbox for the surface-based analysis of structural MRIs. It can help researchers save time to process large datasets, reduce errors in the cumbersome setting of parameters, and provide convenient tools for statistical analysis and reporting of results. However, the current toolbox only works on cross-sectional datasets. The analysis of longitudinal datasets will be programmed in future version. SPAMRI is made freely available on the NITRC website (https://www.nitrc.org/projects/longzhiliangmri/).

## Data Availability Statement

The datasets presented in this study can be found in online repositories (https://openneuro.org/datasets/ds000201).

## Ethics Statement

The study involving human participants was reviewed and approved by the Regional Ethics Review Board of Stockholm (2012/1870-32). The patients/participants provided their written informed consent to participate in this study.

## Author Contributions

ZL designed this research, programmed the SPAMRI toolbox, validated this toolbox, and drafted the manuscript.

## Funding

This work was supported by the National Natural Science Foundation of China (81901725).

## Conflict of Interest

The author declares that the research was conducted in the absence of any commercial or financial relationships that could be construed as a potential conflict of interest.

## Publisher's Note

All claims expressed in this article are solely those of the authors and do not necessarily represent those of their affiliated organizations, or those of the publisher, the editors and the reviewers. Any product that may be evaluated in this article, or claim that may be made by its manufacturer, is not guaranteed or endorsed by the publisher.
