# Supplementary material for: SPAMRI: A MATLAB Toolbox for Surface-Based Processing and Analysis of Magnetic Resonance Imaging
Source: Front Hum Neurosci. 2022 Jul 7;16:946156. doi: 10.3389/fnhum.2022.946156 (PMC9301123; doi:10.3389/fnhum.2022.946156)
Supplement: Supplementary file 1 [file Data_Sheet_1.docx]

Supplementary Material

**RFT procedure in SurfStat**

The RFT procedure in SPAMRI was programmed on the basis of SurfStat functions. Thus, the RFT-thresholded statistical maps of SurfStat are comparable with those generated by using SPAMRI tools. The codes used in SurfStat are as follows:

avf_l = SurfStatListDir('fsaverage/surf/lh.pial');

avf_r = SurfStatListDir('fsaverage/surf/rh.pial');

avsurf = SurfStatReadSurf( [avf_l, avf_r] );% load a pial surface.

Y =SurfStatReadData(‘cortical thickness map’);% load thickness map

Age = term( Age );% a label with Old or Young

M = 1 + Age;

slm = SurfStatLinMod( Y, M, avsurf );

slm = SurfStatT( slm, Age.Old - Age.Young ); % one-tailed test of Old > Young

pval = SurfStatP( slm, mean(Y)~=0 );

SurfStatView( pval, avsurf, 'Old > Young' );

slm = SurfStatT( slm, Age.Young - Age.Old ); % one-tailed test of Old < Young

pval = SurfStatP( slm, mean(Y)~=0 );

SurfStatView( pval, avsurf, 'Old < Young').

The default cluster-forming threshold of p in SurfStat is equal to 0.001. As shown in Figure 7 and Figure 8, the cluster-wise and vertex-wise RFTs in SurfStat (one-tail test) detected more significant clusters than that in SPAMRI (two-tailed test).
